# New Clinical Classification for Ventricular Free Wall Rupture following Acute Myocardial Infarction

**DOI:** 10.1155/2021/1716546

**Published:** 2021-01-02

**Authors:** Wei Gong, Shaoping Nie

**Affiliations:** ^1^Emergency & Critical Care Center, Beijing Anzhen Hospital, Capital Medical University, Beijing, China; ^2^Beijing Institute of Heart, Lung, and Blood Vessel Diseases, Beijing, China

## Abstract

Ventricular free wall rupture (FWR) is a catastrophic complication after acute myocardial infarction (AMI). However, patients with FWR die of cardiac tamponade secondary to intrapericardial hemorrhage that can be treated if properly diagnosed. Unfortunately, FWR was still not diagnosed and classified quickly and accurately. The aim of this study was to present a new clinical classification for FWR. Seventy-eight patients with FWR after STEMI were enrolled in the study. We classified FWR, according to clinical situations after onset, into the cardiac arrest type, unstable type, and stable type. The cardiac arrest type was the most common type, accounting for about 83.3%. 90.8% of patients of this type were complicated with electromechanical dissociation at the time of FWR onset, and 100% of patients of this type died in the hospital. The unstable type was characterized by sudden clinical condition changes with moderate/massive pericardial effusion. In this study, 9.0% of patients were diagnosed as the unstable type. The average time from onset to death was 4.5 hours. This period was the “golden time” to rescue such patients. The stable types usually have stable hemodynamics, but may worsen, requiring rigorous detection of pericardial effusion and vital signs. In this study, 7.7% of patients were diagnosed as the stable type, and 83.5% of them survived in the hospital. The new clinical classification provides a basis for clinical diagnosis and treatment of FWR. The clinical application of the new classification is expected to improve the prognosis of FWR patients.

## 1. Introduction

Ventricular free wall rupture (FWR) is one of the main causes of death in patients with acute myocardial infarction (AMI) [1–3]. Nevertheless, investigation in this field has been ignored, and surprisingly, it has been generally accepted that FWR following AMI is a hopeless situation. However, patients with FWR die of cardiac tamponade secondary to intrapericardial hemorrhage that can be treated if properly diagnosed [4, 5]. Unfortunately, FWR was still not diagnosed and classified quickly and accurately.

The onset of FWR is sudden, with various forms, and is extremely dangerous. Rapid diagnosis and reasonable initial management are the key to the treatment of FWR. Therefore, a clinical classification that can guide the initial treatment is very important for FWR. However, the existing classifications were mostly based on autopsy or surgery; these anatomical-based classifications were of little help for the early assessment and initial treatment of FWR. Becker and colleagues identified 3 morphological types of FWR. Type I rupture is characterized as an abrupt, slit-like myocardial tear and corresponds to the acute phase of AMI (<24 hours). In the type II rupture, an area of myocardial erosion is evident, indicating a slowly progressive tear. The type III rupture has marked thinning of the myocardium and perforation in the central portion of aneurysm, which typically occurs during the late phase of AMI (>7 days) [6]. Purcaro et al. classified FWR into six pathologic types according to autopsy or surgery [7]. Haddadin et al. divided the FWR into oozing type and blow-out type based on the observation of the rupture site during the operation [8]. The new classification is based on the clinical manifestations of patients, which can be quickly classified while diagnosing FWR, providing a basis for treatment of FWR.

## 2. Methods

### 2.1. Study Design and Patient Selection

This is a retrospective study. A total of 6,712 consecutive patients who presented with ST-segment elevation myocardial infarction (STEMI) admitted to Beijing Anzhen Hospital (Beijing, China), from January 2010 to November 2017, were analyzed, and 78 patients with FWR after STEMI were enrolled in the study. STEMI was defined according to the following criteria: ongoing ischemic symptoms, typical rise or fall in cardiac biomarkers, and a new ST elevation in two or more contiguous leads (lead V1~V3 elevation of at least 0.2 mV or the remaining lead elevation of at least 0.1 mV) or new developed left bundle-branch block pattern^11^.

The diagnosis of FWR was based on surgery results or clinical manifestations and results of examination (pericardiocentesis and echocardiography). The cardiac arrest type was defined as an instantaneous circulatory collapse with massive pericardial effusion and cardiac tamponade. The unstable-type FWR was characterized by sudden clinical condition changes (such as syncope, transient arrhythmia, transient gatism, transient EMD, and sudden angina pectoris) with moderate/massive pericardial effusion. The diagnosis of this type of FWR needs to meet an additional condition: pericardiocentesis showing bloody fluid or no/mild pericardial effusion 24 hours ahead of FWR onset. Stable-type FWR was defined as a patient with hypotension with moderate/massive pericardial effusion and diagnostic pericardiocentesis showing bloody fluid.

The study was approved by the ethics committee of Beijing Anzhen Hospital. All procedures followed were in accordance with the Helsinki Declaration of 1975, as revised in 2008.

### 2.2. Data Collection

Baseline clinical characteristics (demographics, medical history, and presenting features) were collected from the medical records of the recruited patients. The parameters of the blood test were the first test results of the patients after admission.

### 2.3. Statistical Analysis

Continuous variables were expressed as means and standard deviations (SD) or median (interquartile range) and compared by analysis of variance. Categorical variables were presented as frequencies and percentages. For comparisons of the distributions of variables between groups, chi-square or Fisher exact tests were used. *P* < 0.05 was considered to be statistically significant. Statistical analysis was performed with IBM SPSS Statistics, Version 22.0 (IBM Corp., Armonk, NY, USA).

## 3. Results

### 3.1. Baseline Patient Characteristics

Seventy-eight patients with FWR after STEMI were enrolled in the study. We classified FWR, according to clinical situations after onset, into cardiac arrest type (*n* = 65), unstable type (*n* = 7), and stable type (*n* = 6). Baseline patient characteristics are shown in Table 1.

### 3.2. Cardiac Arrest Type

The cardiac arrest type was the most common FWR type, which is the sudden rupture with massive hemorrhage into the pericardial cavity that is followed by sudden loss of consciousness and cardiac arrest. In this study, we found that 83.3% of FWR patients were of the cardiac arrest type. This type of patients usually has an exact rupture time, and 90.8% of patients were complicated with electromechanical dissociation (EMD) at the time of FWR onset (Table 2). The time from onset to circulatory collapse of this type of patient was extremely short, and there is still no effective treatment. This type of patient requires urgent pericardial puncture drainage to stabilize hemodynamics and then urgent surgical treatment. In this study, 100% of cardiac arrest-type patients died in the hospital (Table 2).

### 3.3. Unstable Type

The unstable type was characterized by sudden clinical condition changes (such as syncope, transient arrhythmia, transient gatism, transient EMD, and sudden angina pectoris) with moderate/massive pericardial effusion. The diagnosis of this type of FWR needs to meet an additional condition: pericardiocentesis showing bloody fluid or no/mild pericardial effusion 24 hours ahead of FWR onset (Table 2). In this study, 7 patients of this type were diagnosed, and 6 of them died in the hospital (Table 2). The average time from onset to death was 4.5 hours. This period was the “golden time” to rescue such patients. Unfortunately, only one patient of this type underwent surgery in the study. We recommended that this type of patients should be operated urgently.

### 3.4. Stable Type

The stable type was defined as a patient with hypotension with moderate/massive pericardial effusion and diagnostic pericardiocentesis showing bloody fluid. This type of patients usually has stable hemodynamics, but may worsen, requiring rigorous detection of pericardial effusion and vital signs. In this study, 6 patients were diagnosed as this type, and 83.5% of them survived in the hospital (Table 2).

## 4. Discussion

In this study, we presented a new clinical classification for FWR and analyzed the characteristics of each type. This classification provides a basis for clinical diagnosis and treatment of FWR.

In previous studies, the diagnosis of FWR mainly depended on autopsy, and the incidence of FWR after AMI ranges from 0.5% to 6.2% [2, 9, 10]. The autopsy rate varied in different studies, which is an important reason for the different incidence of FWR in different studies. We hold the idea that the incidence of FWR may be underestimated; for instance, patients with pericardial effusion after STEMI may be caused by FWR, because of the low autopsy rate. Therefore, a new diagnosis and classification method based on clinical manifestations is urgently needed. FWR complicating STEMI has been generally considered a hopeless situation [1–3]. However, patients with FWR die of cardiac tamponade secondary to intrapericardial hemorrhage that can be treated if properly diagnosed. Some previous studies showed that urgent surgery plays a key role in the management of FWR, and sutureless repair can be a viable treatment option [11–13]. In fact, only 21.1% of patients diagnosed with cardiac rupture underwent surgery [2]. We believe that the following reasons may cause the low rate of surgical treatment: most FWR patients occurred with sudden death and had no time window for surgical treatment; clinicians lack understanding of FWR, are unable to diagnose and classify FWR in time, and do not master the indications of urgent surgery. The existing classifications were mostly based on autopsy or surgery; these anatomical-based classifications were of little help to the early assessment and initial treatment of FWR. Becker and van Mantgem identified 3 morphological types of FWR [6]. Purcaro et al. classified FWR into six pathologic types according to autopsy or surgery [7]. Haddadin et al. divided the FWR into an oozing type and a blow-out type based on the observation of the rupture site during the operation [8]. The new classification is based on the clinical manifestations of patients, which can be quickly classified while diagnosing FWR, providing a basis for treatment of FWR.

The cardiac arrest type of patients usually has a sudden onset and a very poor prognosis. This type of patient requires urgent pericardial puncture drainage to stabilize hemodynamics and then urgent surgical treatment, but the effect is still very poor. In this study, 100% of cardiac arrest-type patients died in the hospital. Urgent and rapid interventional repair methods are expected to provide new possibilities for the treatment of this type of patient. The unstable type was characterized by sudden clinical condition changes with moderate/massive pericardial effusion. Patients of this type usually survive for a certain period after onset, but the hemodynamics of these patients were unstable and gradually worsen. In this study, the average time from onset to death was 4.5 hours. This period was the “golden time” to rescue such patients. If surgery is urgently performed during this period, the survival rate of patients may be greatly improved. Unfortunately, only one patient of this type underwent surgery in the study. We recommended that the patients should be operated urgently. Sutureless repair can be a viable treatment option [12]. If necessary, pericardiocentesis and drainage of effusion should be done or a mechanical assistant device should be used to maintain hemodynamics, so as to win time for surgery. Stable types usually have stable hemodynamics, but may worsen, requiring rigorous detection of pericardial effusion and vital signs. For diagnostic pericardiocentesis, a drainage tube should be left. It is not recommended to drain pericardial effusion when the hemodynamics is stable and there is no pericardial tamponade. In case of hemodynamic instability and cardiac tamponade, it is recommended to drain the pericardial effusion through an indwelling drainage tube. After drainage of the pericardial effusion, if the patient's hemodynamics is stable, it is recommended to continue to observe closely. If the patient's hemodynamics is still unstable and the pericardial tamponade reappears quickly, emergency surgery should be performed. In this study, 83.5% of this type of patients survived in the hospital based on the above treatment strategies.

Several limitations exist in the study. First of all, this was a retrospective study; therefore, some information is subject to certain inherent limitations and potential biases, including collection of incomplete or missing information and so on. Secondly, FWR is a rare clinical condition and thus involves a limited number of cases. A further limitation represents undetected cases of “minor” FWR that might alter the overall picture of our results; incomplete ruptures are easy to miss.

## 5. Conclusion

The new clinical classification provides a basis for clinical diagnosis and treatment of FWR. The clinical application of the new classification is expected to improve the prognosis of FWR patients.

## Figures and Tables

**Table 1:** Baseline patient characteristics.

Variables	All FWR (*n* = 78)	Cardiac arrest type (*n* = 65)	Unstable type (*n* = 7)	Stable type (*n* = 6)
Age (years)	73.0 [67.5, 79.0]	73.0 [69.5, 79.0]	75.0 [59.0, 77.0]	55.5 [51.8, 63.5]
Female	43 (55.1)	37 (56.9)	3 (42.9)	3 (50.0)
Smoking	24 (30.8)	22 (33.8)	1 (14.3)	1 (16.7)
Alcohol	14 (17.9)	12 (18.5)	0 (0.0)	2 (33.3)
Prior hypertension	47 (60.3)	37 (56.9)	6 (85.7)	4 (66.7)
Prior diabetes	23 (29.5)	21 (32.3)	1 (14.3)	1 (16.7)
Prior MI	6 (7.7)	6 (9.2)	0 (0.0)	0 (0.0)
Anterior MI	51 (65.4)	40 (61.5)	6 (85.7)	5 (83.3)
Time from onset to admission (hours)	6.0 [3.0, 11.3]	6.0 [3.0, 12.5]	10.0 [2.0, 12.0]	4.0 [1.8, 6.0]
SBP (mm hg)	115.0 [103.0, 126.5]	120.0 [105.0, 130.0]	105.0 [95.0, 110.0]	115.0 [90.5, 125.3]
Heart rate (bpm)	79.5 [65.0, 92.3]	78.5 [63.3, 91.3]	80.0 [70.0, 110.0]	76.5 [61.5, 99.3]
Killip class III or IV	18 (23.1)	16 (24.6)	1 (14.3)	1 (16.7)
WBC (1,000/mm^3^)	11.3 ± 3.2	11.2 ± 3.2	11.6 ± 2.8	11.9 ± 4.6
Hemoglobin (g/L)	133.0 [121.0, 140.0]	132.0 [124.0, 139.0]	137.0 [112.0, 153.0]	136.0 [117.0, 151.3]
Platelet count (1,000/mm^3^)	211.5 [170.0, 248.5]	215.0 [170.0, 251.0]	218.0 [182.0, 247.0]	172.5 [144.5, 227.0]
Aspirin+thienopyridine	72 (92.3)	61 (93.8)	5 (71.4)	6 (100.0)
Anticoagulants	71 (91.0)	60 (92.3)	5 (71.4)	6 (100.0)
ACEI/ARB	24 (30.8)	20 (30.8)	1 (14.3)	3 (50.0)
*β*-Blockers	41 (52.6)	33 (50.8)	3 (42.9)	5 (83.3)
Statin	65 (83.3)	56 (86.2)	4 (57.1)	5 (83.3)
Primary PCI	19 (24.3)	12 (18.5)	3 (42.9)	4 (66.7)
Thrombolysis	6 (7.7)	5 (7.7)	0 (0.0)	1 (16.7)

Data given as *n* (%), mean ± SD, or median (IQR). ACEI: angiotensin-converting enzyme inhibitor; ARB: angiotensin receptor blocker; IQR: interquartile range; MI: myocardial infarction; PCI: percutaneous coronary intervention; SD: standard deviation; SBP: systolic blood pressure; WBC: white blood cell counts.

**Table 2:** The new FWR clinical classification.

	Cardiac arrest type (83.3%)	Unstable type (9.0%)	Stable type (7.7%)
Main criteria	Instantaneous circulatory collapse with massive pericardial effusion and cardiac tamponade	Sudden clinical condition changes^∗^ with moderate/massive pericardial effusion	Hypotension with moderate/massive pericardial effusion
Additional conditions for diagnosis of FWR	None	Pericardiocentesis showing bloody fluid or no/mild pericardial effusion 24 hours ahead of FWR onset	Pericardiocentesis showing bloody fluid
Manifestations and signs	(i) Sudden loss of consciousness (ii) Cardiac arrest	(i) Patients with sudden changes in the clinical condition (such as syncope, transient arrhythmia, transient gatism, transient EMD, and sudden angina pectoris) (ii) Most patients with cardiac tamponade (jugular venous distention, muffled heart sounds, and a paradoxical pulse)	(i) Persistent hypotension or refractory angina pectoris or restlessness or no apparent symptom etc. (ii) Hypotension (iii) Some patients with cardiac tamponade (jugular venous distention, muffled heart sounds, and a paradoxical pulse)
Hemodynamic	(i) Sudden collapse	(i) Unstable and gradual worsening	(i) Stable, but may worsen
Pericardial effusion	(i) Massive pericardial effusion (ii) No/mild pericardial effusion ahead of FWR onset	(i) Moderate/massive pericardial effusion (ii) No/mild pericardial effusion ahead of FWR onset	(i) Moderate/massive pericardial effusion (ii) Pericardial effusion increased gradually
EMD	Most patients with EMD (59/65)	Some patients with EMD (3/7)	None
Get accurate rupture time	Yes	Yes	No
Hospital mortality	100%	85.7%	16.7%

^∗^Syncope, transient arrhythmia, transient gatism, transient EMD, sudden angina pectoris, etc. EMD: electromechanical dissociation; FWR: free wall rupture.

## Data Availability

The data used to support the findings of this study are available from the corresponding author upon request.
